# Supplementary material for: Decoding early stress signaling waves in living plants using nanosensor multiplexing
Source: Nat Commun. 2024 Apr 5;15:2943. doi: 10.1038/s41467-024-47082-1 (PMC10997764; doi:10.1038/s41467-024-47082-1)
Supplement: Supplementary file 1 — Supplementary Information [file 41467_2024_47082_MOESM1_ESM.pdf]

Supporting Information

Decoding early stress signaling waves in living plants using nanosensor multiplexing

Mervin Chun-Yi Ang[1#], Jolly Madathiparambil Saju[2#], Thomas K. Porter[3], Sayyid Mohaideen[1], Sarangapani Sreelatha[2], Duc Thinh Khong[1], Song Wang[1], Jianqiao Cui[3], Suh In Loh[1], Gajendra Pratap Singh[1], Nam-Hai Chua[1,2], Michael Strano[1,3*], Sarojam Rajani[1,2*]

[1]Disruptive & Sustainable Technologies for Agricultural Precision IRG, Singapore-MIT Alliance for Research and Technology, 1 CREATE Way, #03-06/07/08 Research Wing, Singapore 138602, Singapore

[2]Temasek Life Sciences Laboratory Limited, 1 Research Link National University of Singapore, Singapore 117604, Singapore

[3]Department of Chemical Engineering, Massachusetts Institute of Technology, 77 Massachusetts Avenue, Cambridge, MA 02139, USA

*Corresponding authors' email address: rajanis@tll.org.sg, and strano@mit.edu

[#] These authors contributed equally to this work.

SUPPLEMENTARY DISCUSSION

Selectivity screening of SA aptamer wrapped SWNT (S5) with plant analytes

Upon screening with the same list of plant hormone analytes as shown in Figure 1d, we found S5 to be relatively inert to all plant hormones with the exception of IAA, which had a moderate quenching response of 18% (Supplementary Figure 2c). We posit that the lack of SA binding of the aptamer could be due to the disruption of aptamer conformation in the process of ultra-sonication during SWNT suspension, hence reducing its binding affinity. Interestingly, we discovered that S5 exhibits increasing binding affinity to SA, upon lowering of the excitation laser wavelength from 750 to 600 nm (Supplementary Figure 2d), resulting in an enlarged SWNT fluorescence quenching response. At t = 50 min, the fluorescence quenching measured after addition of 100 µM of SA, using a 600-nm excitation laser wavelength reached 80%. However, excitation lasers with lowered wavelengths overlap with the chlorophyll auto-fluorescence in plants, giving rise to high background fluorescence captured by the nIR camera. Hence, excitation lasers with longer wavelength at 785 nm and 830 nm are typically more compatible for experimental studies with living plant samples. In contrast, from the 2D excitation-emission map of S3 before and after introduction of SA (Supplementary Figure 3a-b), similar magnitude of fluorescence quenching is observed for all SWNT chiralities across all excitation laser wavelengths from 500 to 800 nm. Hence, we find S3 to be the more suitable plant nanobionic sensor for SA.

Surface coverage of polymer wrapped SWNTs (S1-S4)

SWNT nIR fluorescence is known to quench in the presence of riboflavin for a variety of corona phases[1]. The magnitude of nIR fluorescence quenching upon addition of riboflavin is also

correlated with the surface coverage of the particular corona phase, with smaller magnitude of fluorescence quenching indicative of a more tightly packed corona[2]. S1 and S2 have Pz and Pm co-monomers with para-linkages forming co-polymers that are highly rigid and rod-like while S3 and S4 have meta-linkages conferring more flexibility in the polymer structure[3] (Supplementary Figure 4a). When the riboflavin molecular probe was added, S1 and S2 have a smaller nIR fluorescence quenching magnitudes of 7.0% and 12.8% respectively compared to S3 and S4 with nIR fluorescence quenching magnitudes of 26.0% and 21.9% respectively (Supplementary Figure 4b). This is likely because S1 and S2 are capable of strong inter-chain π-π interactions that enable them to pack closer together on the SWNT surface, leaving less accessible surface area for analyte adsorption on the SWNT surface (Supplementary Figure 4c). As a result, they have relatively inert corona phases. Comparatively, S3 and S4 have enhanced conformational freedom that leaves larger surface area on the SWNT surface available for analyte adsorption. From the CoPhMoRe screening results, it is evident that both S3 and S4 could bind with different plant hormone analytes, resulting in fluorescence intensity modulations. Out of the two, the Pz co-monomer in S3 promoted better selectivity for SA, compared to Pm in S4, which had non-specific binding with a number of plant hormones.

Detailed method for SA concentration calculation using nanosensor calibration curve

The detailed method for SA concentration calculation is explained using the pipecolic acid experiment as an example. Firstly, the average fluorescence intensity is integrated over the entire sensor spot area harbouring many cells as shown in the brightfield image of the leaf (Supplementary Figure 8a). After sensor infiltration, the initial fluorescence intensity (I_0) represents the total basal SA levels present in the cells within the spot area prior to stress (t = 0 h), as illustrated by the false-color sensor intensity maps of the imaged leaf area

(Supplementary Figure 8b). Representative false-color intensity maps are also shown for the same imaged leaf areas at t = 2 h, 4 h and 6 h post treatment with pipecolic acid. By integrating the intensity maps obtained over the 6 h time period, we can obtain fluorescence intensity time curves of the imaged leaf areas. The sensor fluorescence intensity changes ($I - I_0$) in the same population of cells are calculated using I_0 as normalization. The normalized intensity time curves (Supplementary Figure 8c) will have an initial intensity of "1". We then focus on detecting the normalized fluorescence intensity deviation post stress, which is indicative of overall amount of SA produced post stress within the sensor spot area. By taking an intensity ratio between the SA sensor and reference sensor which is non-responsive to SA over time (Supplementary Figure 8d), we further account for other sensor fluctuations unrelated to SA signaling *in planta* post stress. The normalized intensity ratios are then averaged across independent biological replicates. Using ImageJ software, SA concentration map images (Supplementary Figure 8e) can be converted from the respective false-color intensity maps by applying the SA sensor calibration curve at Figure 1g of $\frac{I_0 - I}{I_0} = A \times \frac{[SA]}{[SA]+K_D}$, where A = 0.40558 and K_D = 31.421 µM. The normalized intensity ratio time curves can also be converted to SA concentration time curves with the same sensor calibration equation (Supplementary Figure 8f). This method allows us to precisely calculate the change in SA over the 6 h period post pipecolic acid treatment within the population of cells in the imaged area *in planta* and has been used consistently to calculate SA concentrations in all time course experiments reported in this paper.

H_2O_2 Waveform and SA Model

We propose the following general chemical mechanism to describe the stress-dependent H_2O_2 and SA signatures observed in Figure 7a-d:

$$A + P \xrightarrow{k} A + A, \tag{S1}$$

$$A \xrightarrow{k_d} B\ , \tag{S2}$$

$$A + pF \xrightarrow{k_{F_1}} F_1, \tag{S3}$$

$$F_1 \xrightarrow{k_{F_2}} F_2 \xrightarrow{k_{F_3}} \ldots \xrightarrow{k_{F_n}} F_n \xrightarrow{k_S} S. \tag{S4}$$

$$A + pI \xrightarrow{k_I} I, \tag{S5}$$

$$I + pF \xrightarrow{k_r} xF_1. \tag{S6}$$

The following mass balances can be derived:

$$\frac{dA}{dt} = kAP - k_I ApI - k_{F_1} ApF - k_d A, \tag{S7}$$

$$\frac{dP}{dt} = -kAP, \tag{S8}$$

$$\frac{dpF}{dt} = -k_{F_1} ApF - k_r IpF, \tag{S9}$$

$$\frac{dF_1}{dt} = k_{F_1} ApF - k_{F_2} F_1, \tag{S10}$$

$$\frac{dF_2}{dt} = k_{F_2} F_1 - k_S F_2 \tag{S11}$$

$$\frac{dS}{dt} = k_S F_2, \tag{S12}$$

$$\frac{dpI}{dt} = -k_I ApI, \tag{S13}$$

$$\frac{dI}{dt} = k_I ApI - k_r IpF, \tag{S14}$$

with nonzero initial conditions

$$A(0) = A_0, \tag{S15}$$

$$P(0) = P_0, \tag{S16}$$

$$pF(0) = pF_0, \tag{S17}$$

$$pI(0) = pI_0. \tag{S18}$$

All other species are initialized at zero. Equations S7-S14 were numerically simulated in MATLAB using the adaptive stiff ODE solver *ode15s*. Because the exact time of stress perception is unclear, especially for stresses applied over longer times, the model was fitted to data while allowing for a time shift t_0 to approximately match the experimental H_2O_2 waveform peak location. Note that both model-generated H_2O_2 and SA were shifted by the same t_0, so the relative onsets of H_2O_2 and SA in the model were unchanged.

Confocal imaging to elucidate nanosensor movement post infiltration

In Lew, T.T.S. et al, Nature Plants 6 (2020), 404–415, it was observed that the ROS and reference sensors do not mix when infiltrated regions are separated by midvein[4]. To further confirm this, we evaluated the movement of sensors post infiltration by confocal imaging. The H_2O_2 nanosensor was tagged with Cy3 florescent dye and then the sensors were infiltrated into three distinct areas of the leaf that are partitioned by dominant veins of the pak choi leaf in the following manner. The Cy3-tagged H_2O_2 sensor and SA sensor were individually infiltrated in two spots and then the two sensors were mixed and infiltrated into the third spot (Supplementary Figure 10). The intracellular

localization pattern of both the sensors overlaps. We focused on the chloroplast localization of both sensors as it is easy to decipher by confocal. After 5hrs post infiltration, confocal scanning of the three infiltrated spots was performed. The region infiltrated with SA sensor showed no fluorescence from the Cy3-tagged H_2O_2 and conversely the area infiltrated with Cy3-tagged H_2O_2 showed no fluorescence from the SA sensor. The region where both the sensors were mixed and infiltrated, fluorescence from both the sensors could be observed. The above experiment indicate that the infiltrated sensors do not migrate out of their infiltrated regions.

REFERENCES

1 Zhang, J. *et al.* Molecular recognition using corona phase complexes made of synthetic polymers adsorbed on carbon nanotubes. *Nat. Nanotechnol.* **8**, 959-968, doi:10.1038/nnano.2013.236 (2013).

2 Park, M. *et al.* Measuring the Accessible Surface Area within the Nanoparticle Corona Using Molecular Probe Adsorption. *Nano Lett.* **19**, 7712-7724, doi:10.1021/acs.nanolett.9b02647 (2019).

3 Liu, B., Wang, S., Bazan, G. C. & Mikhailovsky, A. Shape-Adaptable Water-Soluble Conjugated Polymers. *J. Am. Chem. Soc.* **125**, 13306-13307, doi:10.1021/ja0365072 (2003).

4 Lew, T. T. S. *et al.* Real-time detection of wound-induced H2O2 signalling waves in plants with optical nanosensors. *Nat. Plants* **6**, 404-415, doi:10.1038/s41477-020-0632-4 (2020).

SUPPLEMENTARY FIGURES

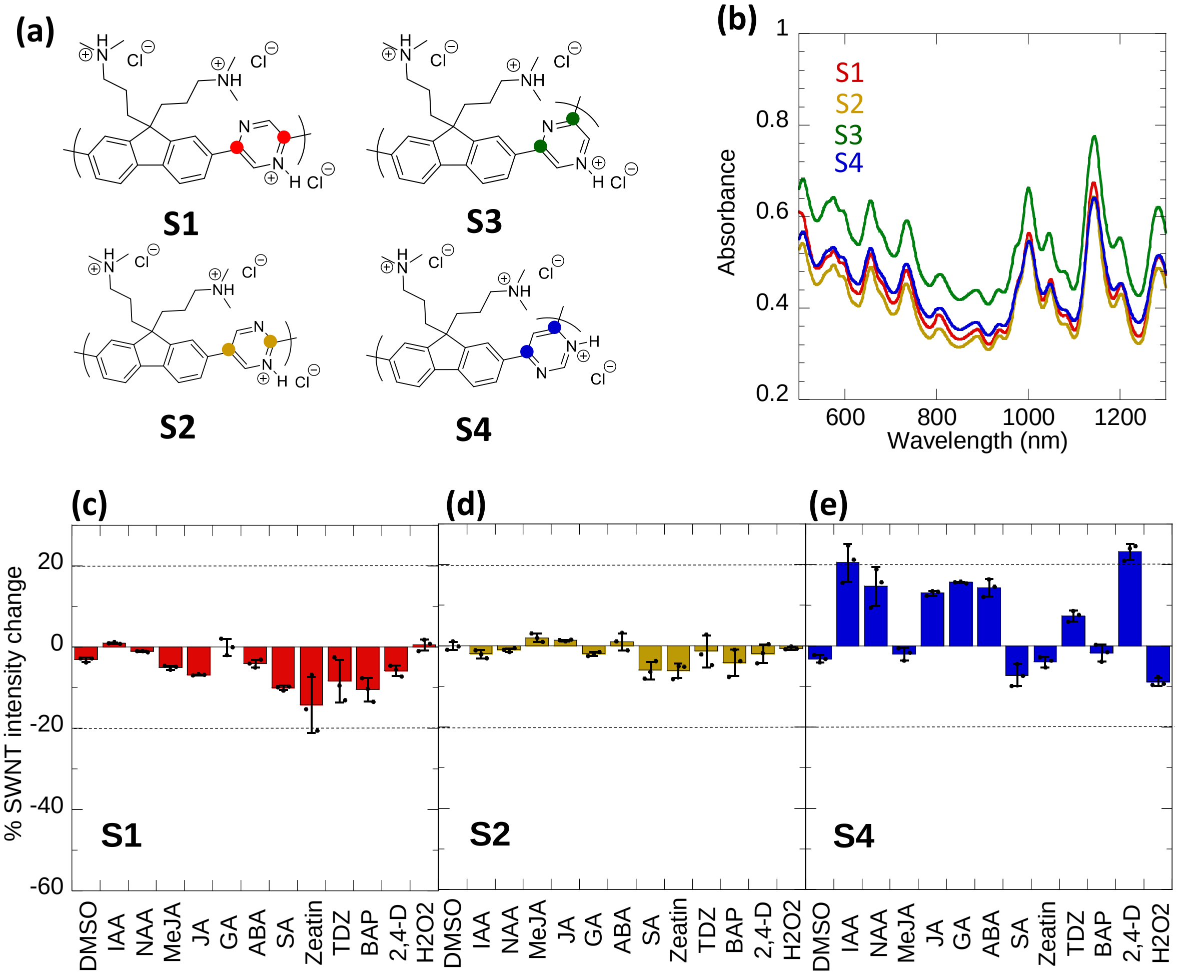

Supplementary Figure 1: (a) Chemical structures of cationic polymer series (S1 – S4) comprising the polyfluorene (PF)-based A-B copolymer backbone; (b) UV−vis−nIR absorption spectra of HiPco-SWNT suspensions: S1 [PF(2,5)PzHCl; red], S2 [PF(2,5)PmHCl; orange], S3 [PF(2,6)PzHCl; green], and S4 [PF(4,6)HCl; dark blue] after 2 h of ultracentrifugation at 153,145g and 5× dilution in deionized (DI) water; [Pz, pyrazine; Pm, pyrimidine; HCl, hydrogen chloride.]; Fluorescence response to 100 µM plant hormone analytes for (c) S1, (d) S2 and (e) S4. Fluorescence quenching or turn-on responses of ±20% (dotted lines) are considered sufficiently significant responses due to analyte binding. Bar graph show the mean values with error bars representing standard deviations from independent experiments (n = 3). Dots represent each data point. DMSO is used as negative control.

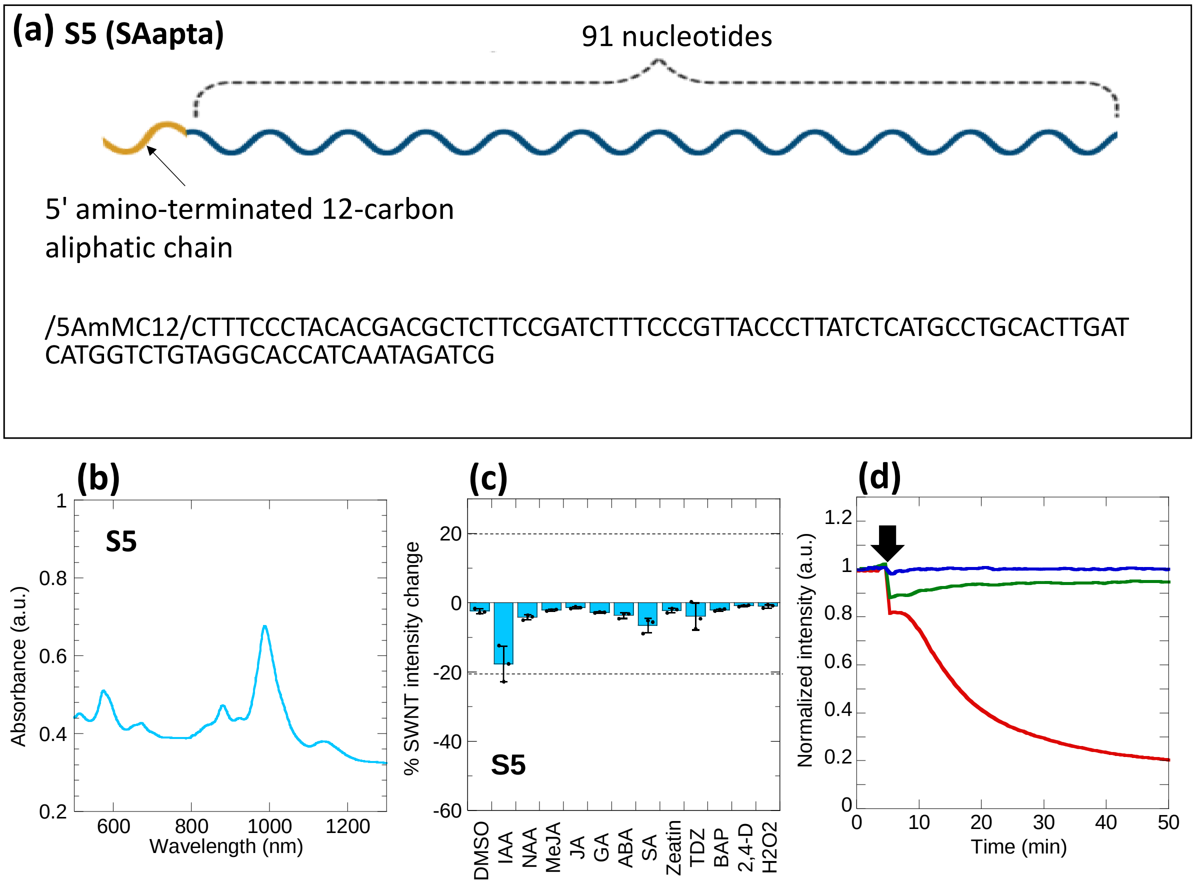

Supplementary Figure 2: (a) Schematic and nucleotide sequence of S5 (SAapta) comprising 91 nucleotides sequence with an amino-modified C12 aliphatic chain at the 5' end; (b) UV−vis−nIR absorption spectra of S5 Comocat (6,5)-SWNT suspension after 2 rounds of 90-min centrifugation at 16,000 g and 9× dilution in DI water; (c) Fluorescence response of S5 to 100 μM plant hormone analytes. Fluorescence quenching or turn-on responses of ±20% (dotted lines) are considered sufficiently significant responses due to analyte binding. Bar graph show the mean values with error bars representing standard deviations from independent experiments (n = 3). Dots represent each data point. Excitation wavelength: 785 nm; (d) Normalized fluorescence intensity of S5 upon addition of SA at t = 5 min (black arrow) when continuously exposed to 600 nm (red), 700 nm (green) and 750 nm (blue) excitation.

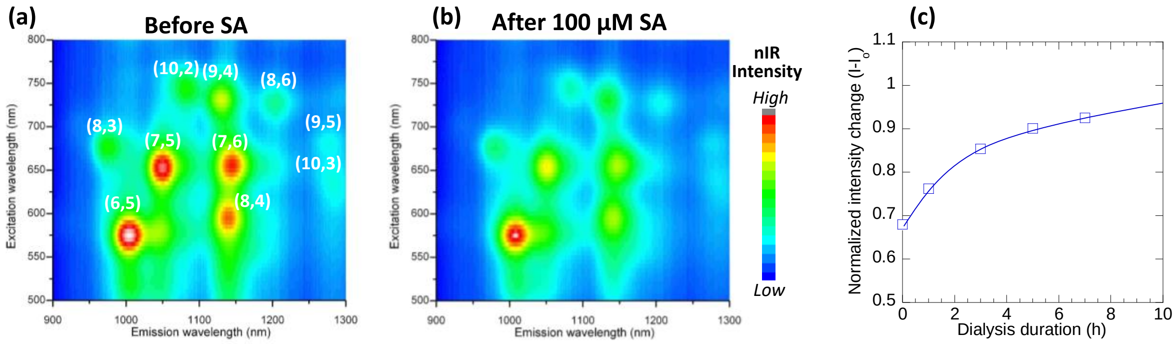

Supplementary Figure 3: 2D excitation-emission map of S3 (a) before and (b) after addition of SA, showing fluorescence quenching across all SWNT chiralities upon SA binding; (c) Demonstration of SA nanosensor reversibility by removing SA from S3 solution over 7 h by dynamic dialysis with 3kDa

MWCO dialysis membrane. Mobile phase: 0.2% DMSO in DI water. Flow rate: 10 mL/min. S3 fluorescence was measured before and after addition of 100 µM SA (t = 0 h), periodically extracted from dialysis tube and measured at t = 1, 3, 5 and 7 h of dialysis.

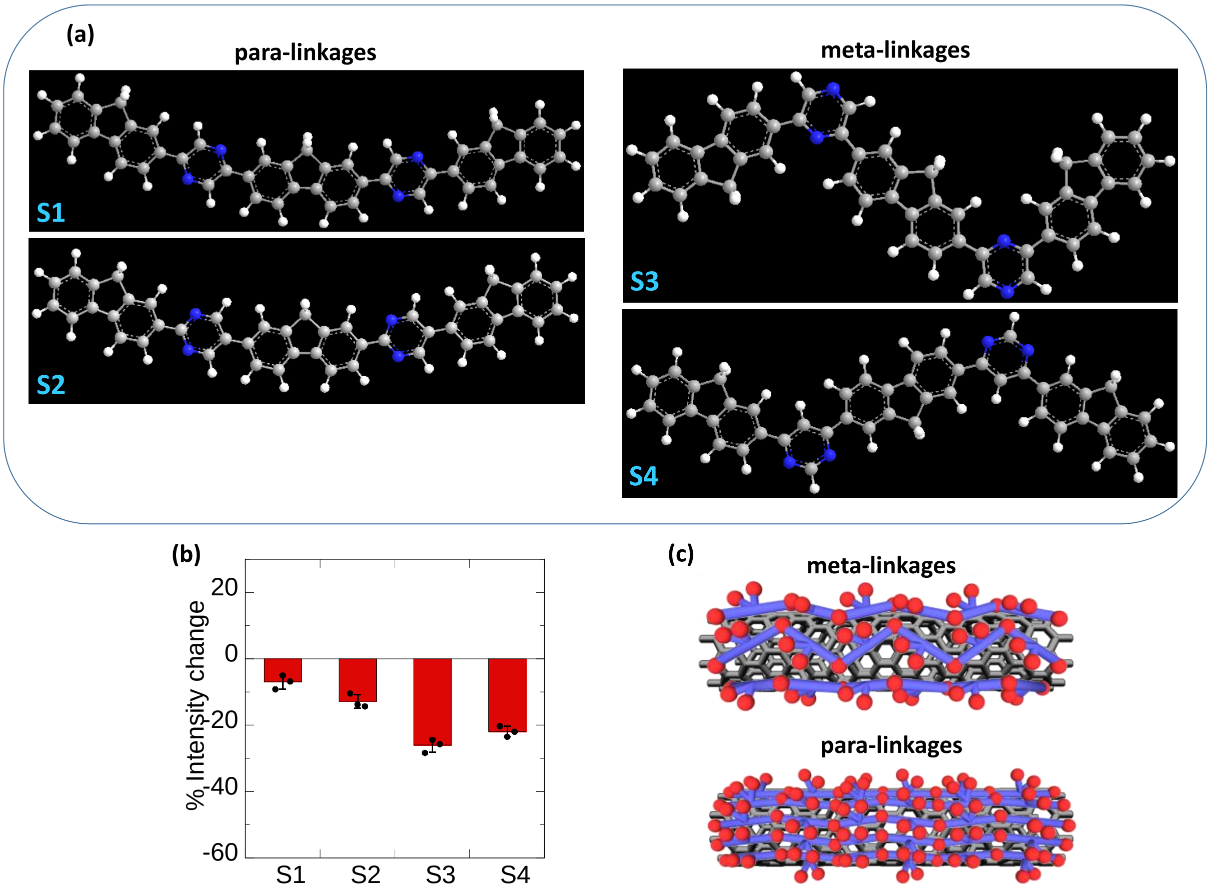

Supplementary Figure 4: (a) PM3 geometry-optimized polymer backbone of S1 to S4 (3 repeat units) indicating enhanced conformational freedom of S3 and S4 polymers with meta-linkages compared to S1 and S2 polymers with para-linkages, possibly conferring improved selectivity for specific analyte binding. Nitrogen: blue; Carbon: dark grey; Hydrogen: light grey; (b) SWNT nIR fluorescence response of S1 – S4 to 10 µM riboflavin, indicative of differing SWNT surface coverage. Bar graph show the mean values with error bars representing standard deviations from independent experiments (n = 3). Dots represent each data point; (c) Schematic of S1-S4 polymer-wrapped SWNTs (grey) with either meta (S3-S4) or para (S1-S2) linkages within the polymer backbone (purple) while cationic side-chains (red) extend out into the solution matrix.

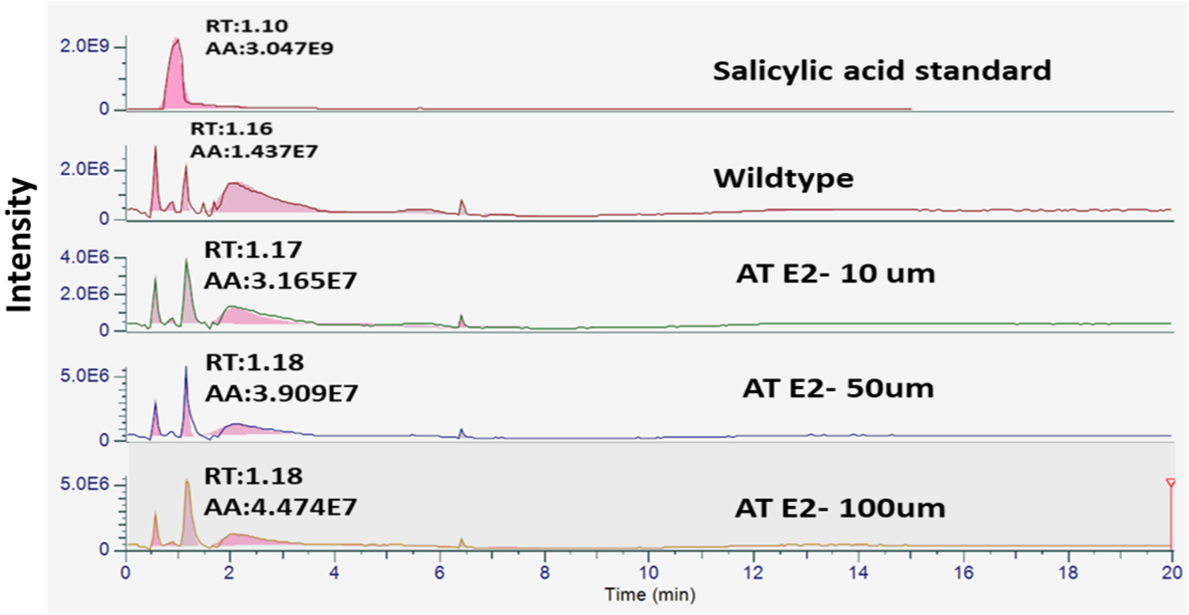

Supplementary Figure 5: Chromatograms of SA standard, leaf sample from wild-type Arabidopsis thaliana and transgenic *XVE::ICS1* Arabidopsis thaliana subjected to 10 µM, 50 µM and 100 µM E2 treatment (top to bottom);

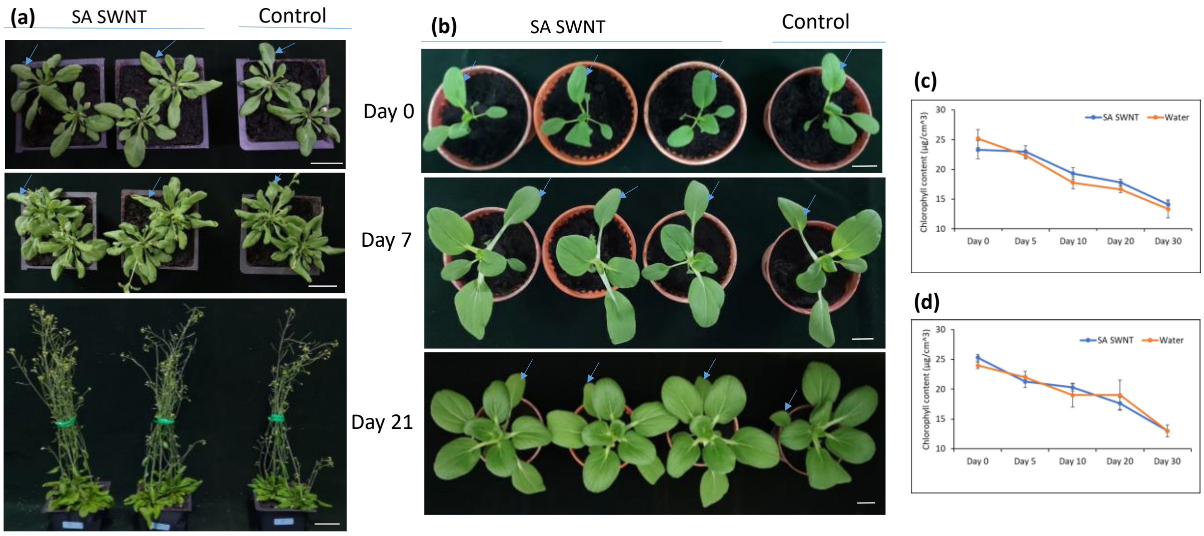

Supplementary Figure 6: SA SWNT biocompatibility evaluation. The SA sensor-infiltrated (a) Arabidopsis and (b) Pak choi plants displayed no difference in overall growth and showed no visible signs of premature senescence compared to control water infiltrated plants; (c,d) No change in the chlorophyll content of the SA sensor-infiltrated leaves when compared to control water infiltrated leaves over a period of 4 weeks (Blue arrows indicate the infiltrated leaf). Error bars represent standard deviations from biologically independent experiments (n = 3). Scale bar = 3 cm.

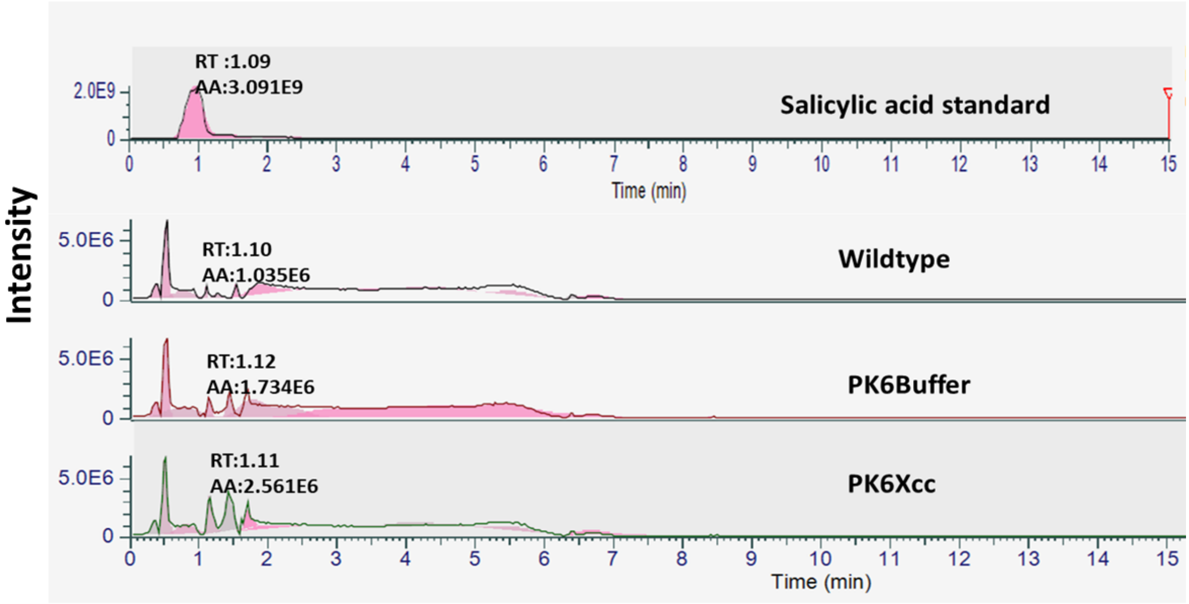

Supplementary Figure 7: Chromatograms of SA standard and pak choi 6 h after buffer infiltration or *Xcc* infection (top to bottom). RT: Retention time; AA: Absolute Area

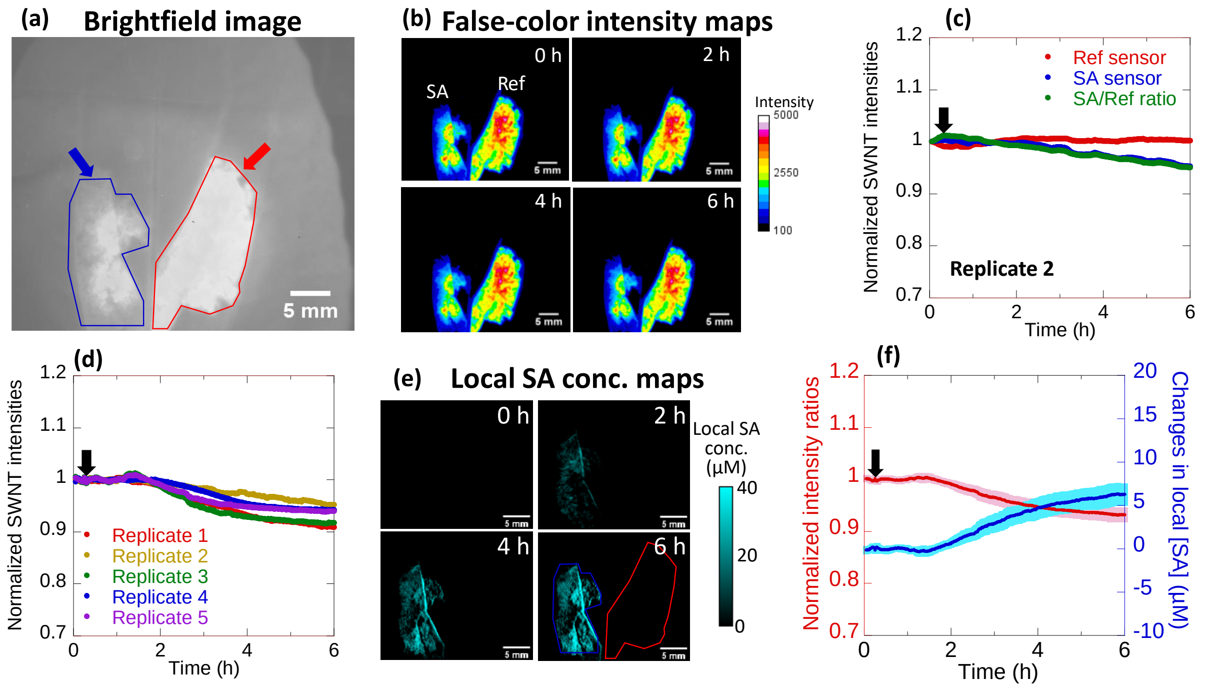

Supplementary Figure 8: Detailed method to calculate the local SA concentration using Pipecolic Acid treatment data as example. (a) Bright-field images of pak choi infiltrated with the reference sensor and SA sensor to the right and left of leaf midvein respectively with sensor areas represented as overlay; (b) False-color images of SA and reference sensor fluorescence before pip treatment (0 h), and after pip treatment (2, 4 and 6 h) showing gradual quenching of SA sensor while reference sensor remains invariant; (c) Time-plot showing the normalized SWNT intensity of reference (red) and SA (blue) sensors as well as the SA/Ref ratio (green) of 1 of the replicates of pak choi undergoing pip treatment. Average intensity of the 1st 20 frames is used for normalization; (d) Normalized SA/Ref ratios obtained for 5 different replicates of pak choi undergoing pip treatment; (e) Change in local SA

concentrations of Pak choi plants before pip treatment (0 h), and after pip treatment (2, 4 and 6 h) derived from false-color intensity maps after applying the SA sensor calibration curve at Figure 1g of $\frac{I_0 - I}{I_0} = A \times \frac{[SA]}{[SA]+K_D}$, where A = 0.40558 and K_D = 31.421 µM; Normalized fluorescence intensity ratios (red) and the corresponding change in the local SA concentration (blue) measured upon 1 mM pipecolic acid (pip) treatment at *t* = 15 min (black arrow). Shaded regions represent standard error across three independent replicates.

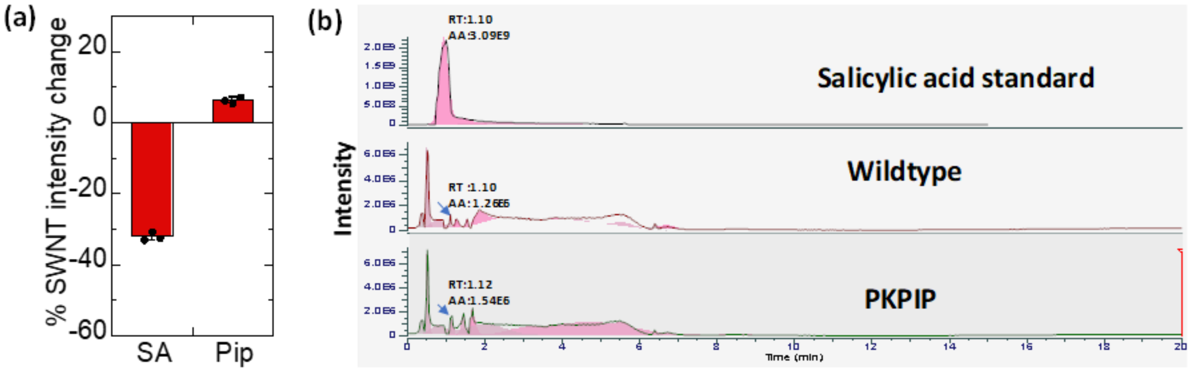

Supplementary Figure 9: (a) *In vitro* fluorescence response of S3 to 100 µM SA shows large quenching response while 100 µM Pip shows negligible response. Bar graph show the mean values with error bars representing standard deviations from biologically independent experiments (n = 3). Dots represent each data point; (b) Chromatograms of SA standard, leaf sample from pak choi sprayed with water and pak choi 6 hrs after Pipecolic acid spray (top to bottom). RT: Retention time; AA: Absolute Area

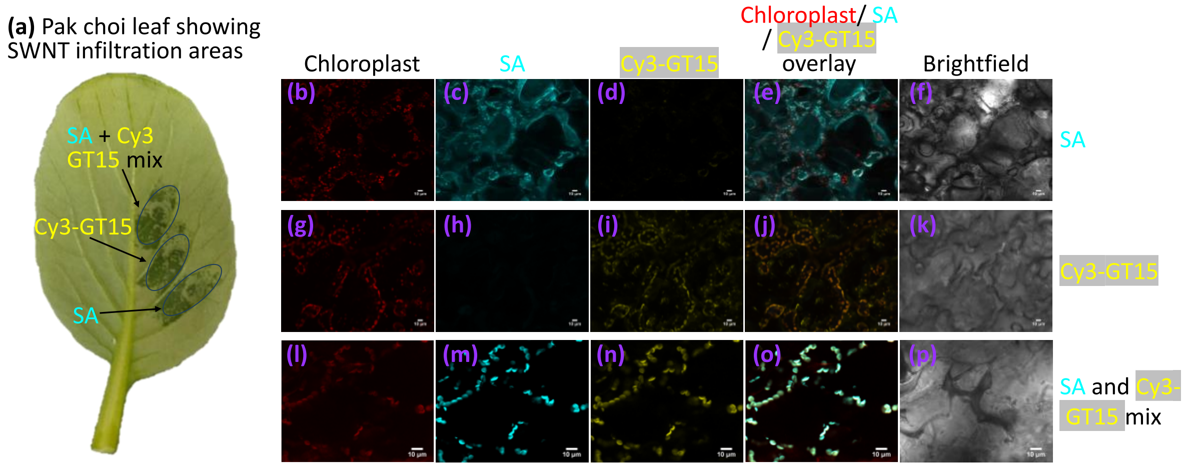

Supplementary Figure 10: (a) Photograph of pak choi leaf illustrating the respective sensor infiltrated regions ; Confocal images of the pak choi leaf infiltrated with (b-f) SA sensor where no fluorescence from Cy3-tagged $(GT)_{15}$-SWNT (ROS sensor) is observed , (g-k) Cy3-tagged $(GT)_{15}$ SWNT (ROS sensor) where no fluorescence from SA sensor is observed and (l-p) mixture of SA sensor and Cy3-tagged $(GT)_{15}$-SWNT (ROS sensor) where fluorescence signal from both SWNTs are observed. Confocal images are representative of at least three independent experiments.

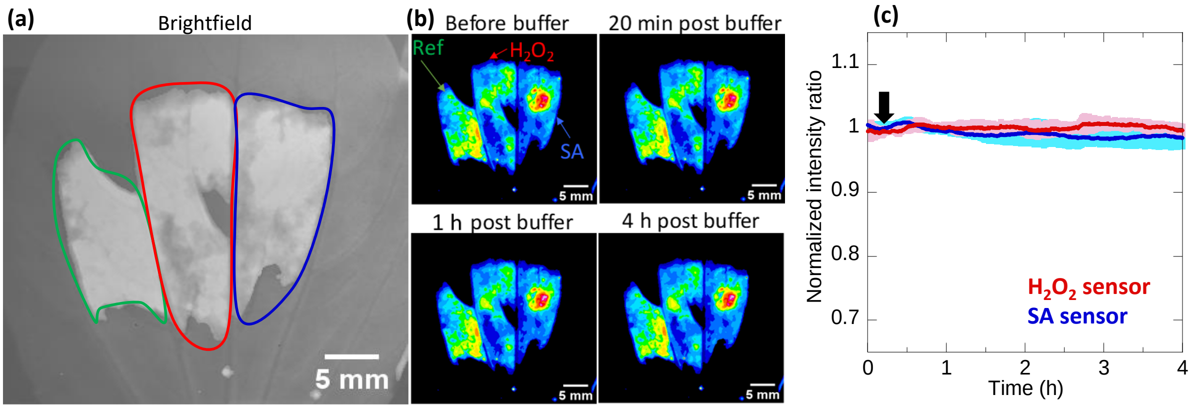

Supplementary Figure 11: (a) Bright-field and (b) corresponding false-color images of pak choi infiltrated with the reference sensor (green), SA sensor (blue), and H_2O_2 sensor (red) under 785 nm laser excitation. Pak choi plants were subjected to blank YGC buffer infiltration; (c) Time-plot of intensity ratios between H_2O_2 and reference sensors (red), and between SA and reference sensors (blue), for YGC buffer infiltrated pak choi plants. Treatment with blank YGC broth occurs at t = 10 min (black arrow). Shaded regions represent standard error across three independent replicates.

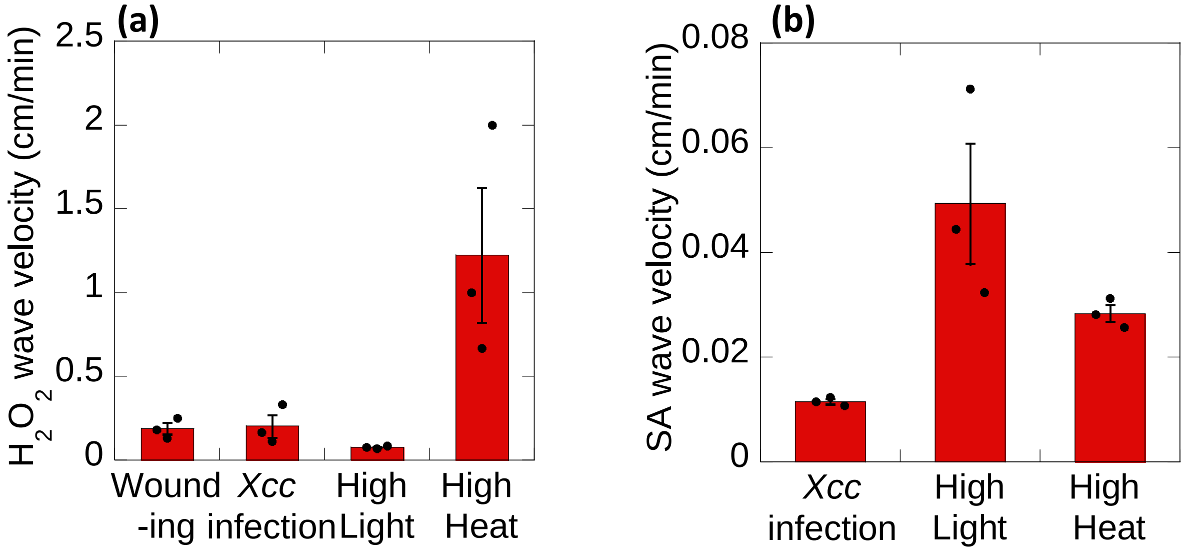

Supplementary Figure 12: (a) Comparison of H_2O_2 wave velocities for pak choi plants that have been subjected to wounding, *Xcc* infection, high light and high heat stresses. (b) Comparison of SA wave velocities for pak choi plants that have been subjected to *Xcc* infection, high light and high heat stresses. Bar graphs show the mean values with error bars representing standard error from independent experiments (n = 3). Dots represent each data point.
