# Supplementary material for: Decoding early stress signaling waves in living plants using nanosensor multiplexing
Source: Nat Commun. 2024 Apr 5;15:2943. doi: 10.1038/s41467-024-47082-1 (PMC10997764; doi:10.1038/s41467-024-47082-1)
Supplement: Supplementary file 3 — Description of Additional Supplementary Files [file 41467_2024_47082_MOESM3_ESM.pdf]

Description of Additional Supplementary Files:

Supplementary Movie 1: Time-lapse video showing H2O2 and SA concentration maps of a pak choi plant subjected to mechanical wounding over 4 h. Pak choi plants were infiltrated with H2O2, SA and reference sensors and placed under 785-nm laser excitation. H2O2 and SA concentration maps are obtained by converting the magnitude of sensor fluorescence quenching to concentrations using their respective sensor calibration curves. The 4 h time-lapse concentration map videos are generated with 60 frames per s, with each frame being 30 s. Mechanical wounding was applied at t = 15 min.

Supplementary Movie 2: Time-lapse video showing H2O2 and SA concentration maps of a pak choi plant subjected to Xcc infection over 4 h. Pak choi plants were infiltrated with H2O2, SA and reference sensors and placed under 785-nm laser excitation. H2O2 and SA concentration maps are obtained by converting the magnitude of sensor fluorescence quenching to concentrations using their respective sensor calibration curves. The 4 h time-lapse concentration map videos are generated with 60 frames per s, with each frame being 30 s. Xcc infection was applied at t = 15 min.

Supplementary Movie 3: Time-lapse video showing H2O2 and SA concentration maps of a pak choi plant subjected to high light treatment over 4 h. Pak choi plants were infiltrated with H2O2, SA and reference sensors and placed under 785-nm laser excitation. H2O2 and SA concentration maps are obtained by converting the magnitude of sensor fluorescence quenching to concentrations using their respective sensor calibration curves. The 4 h time-lapse concentration map videos are generated with 60 frames per s, with each frame being 30 s. High light treatment was applied at t = 15 min for 5 min.

Supplementary Movie 4: Time-lapse video showing H2O2 and SA concentration maps of a pak choi plant subjected to high heat treatment over 4 h. Pak choi plants were infiltrated with H2O2, SA and reference sensors and placed under 785-nm laser excitation. H2O2 and SA concentration maps are obtained by converting the magnitude of sensor fluorescence quenching to concentrations using their respective sensor calibration curves. The 4 h time-lapse concentration map videos are generated with 60 frames per s, with each frame being 30 s. High heat treatment was applied at t = 15 min.
