# Supplementary material for: Decoding early stress signaling waves in living plants using nanosensor multiplexing
Source: Nat Commun. 2024 Apr 5;15:2943. doi: 10.1038/s41467-024-47082-1 (PMC10997764; doi:10.1038/s41467-024-47082-1)
Supplement: Supplementary file 8 — Reporting Summary [file 41467_2024_47082_MOESM8_ESM.pdf]

nature portfolio

Corresponding author(s): Michael S. Strano, Sarojam Rajani

Last updated by author(s): Mar 1, 2024

Reporting Summary

Nature Portfolio wishes to improve the reproducibility of the work that we publish. This form provides structure for consistency and transparency in reporting. For further information on Nature Portfolio policies, see our Editorial Policies and the Editorial Policy Checklist.

Statistics

For all statistical analyses, confirm that the following items are present in the figure legend, table legend, main text, or Methods section.

n/a	Confirmed	
☐	☒	The exact sample size (*n*) for each experimental group/condition, given as a discrete number and unit of measurement
☐	☒	A statement on whether measurements were taken from distinct samples or whether the same sample was measured repeatedly
☐	☒	The statistical test(s) used AND whether they are one- or two-sided<br>*Only common tests should be described solely by name; describe more complex techniques in the Methods section.*
☒	☐	A description of all covariates tested
☐	☒	A description of any assumptions or corrections, such as tests of normality and adjustment for multiple comparisons
☐	☒	A full description of the statistical parameters including central tendency (e.g. means) or other basic estimates (e.g. regression coefficient) AND variation (e.g. standard deviation) or associated estimates of uncertainty (e.g. confidence intervals)
☐	☒	For null hypothesis testing, the test statistic (e.g. *F*, *t*, *r*) with confidence intervals, effect sizes, degrees of freedom and *P* value noted<br>*Give P values as exact values whenever suitable.*
☒	☐	For Bayesian analysis, information on the choice of priors and Markov chain Monte Carlo settings
☒	☐	For hierarchical and complex designs, identification of the appropriate level for tests and full reporting of outcomes
☒	☐	Estimates of effect sizes (e.g. Cohen's *d*, Pearson's *r*), indicating how they were calculated

Our web collection on statistics for biologists contains articles on many of the points above.

Software and code

Policy information about availability of computer code

Data collection	Videos of nanosensor fluorescence images were collected using LightField software. Nanosensor in vitro selectivity screening data were collected using Jobin-Yvon Nanolog-3 spectrometer. Confocal images were acquired with an inverted confocal microscope (SP8, Leica, Germany) using LAS X software. Chlorophyll content of leaves was measured using a CI-710s Spectra Vue (CID-Bioscience USA) leaf spectrometer. LC detection was performed in a targeted selected ion monitoring mode on the Thermo Orbitrap Q–Exactive quadrupole mass spectrometer. Xcalibur software (version 5.0, Thermo) was used to control the instrument and to acquire and process the MS data.
Data analysis	Statistical tests were performed in Kaleidagraph Version 4.5.2. Nanosensor videos, nanosensor images and confocal images were analyzed using ImageJ 1.52p software, as well as MATLAB R2021a. For LCMS analysis, trace finder and free style software (version 5.0, Thermo) was used for pre-processing the raw data and to determine the analyte peak area response relative to external standard.

For manuscripts utilizing custom algorithms or software that are central to the research but not yet described in published literature, software must be made available to editors and reviewers. We strongly encourage code deposition in a community repository (e.g. GitHub). See the Nature Portfolio guidelines for submitting code & software for further information.

Data

Policy information about availability of data

All manuscripts must include a data availability statement. This statement should provide the following information, where applicable:

- Accession codes, unique identifiers, or web links for publicly available datasets
- A description of any restrictions on data availability
- For clinical datasets or third party data, please ensure that the statement adheres to our policy

The authors declare that all data supporting the findings of this study are available within the paper and any raw data can be obtained from the corresponding author on request.

Research involving human participants, their data, or biological material

Policy information about studies with human participants or human data. See also policy information about sex, gender (identity/presentation), and sexual orientation and race, ethnicity and racism.

Reporting on sex and gender	NA
Reporting on race, ethnicity, or other socially relevant groupings	NA
Population characteristics	NA
Recruitment	NA
Ethics oversight	NA

Note that full information on the approval of the study protocol must also be provided in the manuscript.

Field-specific reporting

Please select the one below that is the best fit for your research. If you are not sure, read the appropriate sections before making your selection.

☒ Life sciences ☐ Behavioural & social sciences ☐ Ecological, evolutionary & environmental sciences

For a reference copy of the document with all sections, see nature.com/documents/nr-reporting-summary-flat.pdf

Life sciences study design

All studies must disclose on these points even when the disclosure is negative.

Sample size	Sample sizes were chosen based on the availability of the plants and the variability of preliminary experiments, with a minimum of n = 3 independent biological replicates to ensure reproducibility of the results. They have been explicitly indicated in the respective table and figure legend.
Data exclusions	No data were excluded from the analysis.
Replication	All experiments throughout the entire study were repeated at least three times to check the consistency of the results.
Randomization	All plant samples were randomly selected from a group of healthy plants grown side-by-side to equalize growth conditions as much as possible. For different batches of plants, the plants are measured at the similar growth stage/age.
Blinding	Investigators were not blinded. Experimental conditions in this study were well-controlled such that no observer bias can occur.

Reporting for specific materials, systems and methods

We require information from authors about some types of materials, experimental systems and methods used in many studies. Here, indicate whether each material, system or method listed is relevant to your study. If you are not sure if a list item applies to your research, read the appropriate section before selecting a response.

Materials & experimental systems

n/a	Involved in the study
☒	☐ Antibodies
☒	☐ Eukaryotic cell lines
☒	☐ Palaeontology and archaeology
☒	☐ Animals and other organisms
☒	☐ Clinical data
☒	☐ Dual use research of concern
☐	☒ Plants

Methods

n/a	Involved in the study
☒	☐ ChIP-seq
☒	☐ Flow cytometry
☒	☐ MRI-based neuroimaging

Dual use research of concern

Policy information about dual use research of concern

Hazards

Could the accidental, deliberate or reckless misuse of agents or technologies generated in the work, or the application of information presented in the manuscript, pose a threat to:

No	Yes
☒	☐ Public health
☒	☐ National security
☒	☐ Crops and/or livestock
☒	☐ Ecosystems
☒	☐ Any other significant area

Experiments of concern

Does the work involve any of these experiments of concern:

No	Yes
☒	☐ Demonstrate how to render a vaccine ineffective
☒	☐ Confer resistance to therapeutically useful antibiotics or antiviral agents
☒	☐ Enhance the virulence of a pathogen or render a nonpathogen virulent
☒	☐ Increase transmissibility of a pathogen
☒	☐ Alter the host range of a pathogen
☒	☐ Enable evasion of diagnostic/detection modalities
☒	☐ Enable the weaponization of a biological agent or toxin
☒	☐ Any other potentially harmful combination of experiments and agents

Plants

Seed stocks	Arabidopsis seeds of wild type Columbia-0 (Col-0)and A. thaliana ICS1 mutant (SALK_111380C) was obtained from A. thaliana Biological Resource Centre (ABRC). Pak choi seeds were obtained from Singapore commercial local seed company
Novel plant genotypes	The AtICS1 coding region was PCR-amplified from A. thaliana and then inserted it into the expression cassette of an XVE system vector (PER8-ICS1). This vector was introduced into Agrobacterium tumefaciens strain EHA105 and used to tranform A. thaliana ICS1 mutant plants by floral dip transformation protocol. Transformants were selected on Hygromycin plates and T3 generation seeds were used for all induction experiments.
Authentication	Single insertion homozygous trangenics were selected based on Hygromycin resistance selection till T3 generation
